# Aromatherapy with inhalation effectively alleviates the test anxiety of college students: A meta-analysis

**DOI:** 10.3389/fpsyg.2022.1042553

**Published:** 2023-01-06

**Authors:** Jiage Luan, Mengshu Yang, Yan Zhao, Yu Zang, Ziyu Zhang, Haiying Chen

**Affiliations:** ^1^School of Nursing, Hebei Medical University, Shijiazhuang, China; ^2^The Second Hospital of Hebei Medical University, Shijiazhuang, China

**Keywords:** complementary therapy, essential oils, students, exam anxiety, systematic review

## Abstract

**Objective:**

Test anxiety is one of the common psychological and behavioral problems of college students, which can result in poor academic performance and even academic failure. Aromatherapy has been proposed as a promising method to reduce test anxiety in college students, but its precise efficacy has not been fully confirmed. This meta-analysis evaluated the effects of aromatherapy on the symptoms of test anxiety in college students to serve as a reference for future research and provide more scientific and exact evidence.

**Methods:**

PubMed, The Cochrane Library, Web of Science, Embase, CINAHL, Science Direct, Chinese National Knowledge Infrastructure (CNKI), Chinese Science and Technology Journal Full-Text Database (VIP), and Wanfang Data were electronically searched from inception to June 2022 to identify randomized controlled trials (RCTs) on aromatherapy for treating students’ test anxiety. The Cochrane Risk of Bias Assessment Tool for RCTs was used by two reviewers to critically and independently assess the methodological quality of the included studies. Review Manager 5.4 was used for the meta-analysis. Stata 17.0 was used for sensitivity analysis and Egger’s test.

**Results:**

Seven RCTs included 425 patients, with a moderate risk of bias in the included studies. The meta-analysis found that aromatherapy effectively reduced test anxiety in college students (SMD = −0.67, *p* < 0.01), with high heterogeneity of results (*I*^2^ = 72%). The different types of essential oil devices used in the study are the source of inter-study heterogeneity. Subgroup analysis suggested that most effective methods were aromatherapy with compound essential oils (SMD = −0.91, *p* < 0.01), and using cloths or pads as the essential oil devices (SMD = −1.03, *p* < 0.01). There was no difference between aromatherapy and placebo control groups (SMD = −0.65, *p* = 0.25).

**Conclusion:**

Aromatherapy with inhalation can help relieve test anxiety in college students, but more and higher-quality studies are needed. This study provides a reference for future research designs in terms of the selection of essential oil types and devices and the design of research groups.

**Systematic review registration:**

[https://www.crd.york.ac.uk/prospero/], identifier [CRD42022345767].

## 1. Introduction

Aromatherapy is a common complementary therapy widely used in many countries in recent years (Lee et al., 2012). It uses pure natural essential oils taken from plants, which can have a beneficial effect on the body and mind (Brennan et al., 2022). It has been found to reduce anxiety, depression, and other negative emotions to efficiently improve physical and mental comfort (Li et al., 2022). Aromatherapy regulates mood by connecting olfaction and the limbic system to stimulate the release of endorphins and serotonin (Liu et al., 2022). Endorphins and serotonin have calming, soothing, and relaxing effects that can relieve anxiety (Singewald et al., 2015). Studies have found that essential oils, including lavender, rose, lemon, mint, and rosemary, are effective in reducing anxiety and depression, reducing stress, enhancing memory, and improving cognitive function (McCaffrey et al., 2009; Moss et al., 2018). Malloggi et al. (2022) found that inhalation of lavender essential oil can reduce arousal levels, improve sustained attention, and enhance cognitive function, which are related to the anti-anxiety effects of lavender. Aromatherapy not only promotes calmness and relaxation but also enhances memory and concentration, which is a potential method for relieving college students’ test anxiety (Mei, 2010; Faydali and Çetinkaya, 2018; Han et al., 2018; Karadag and Baglama, 2019; Malloggi et al., 2022).

Tests are key tools for talent assessment and a significant aspect of life for modern college students (Rosenberg and Hamiel, 2021). Influenced by many factors, such as fierce labor market competition, the pressure of college entrance development, and family, test anxiety has become one of the most common psychological and behavioral problems of college students (Quinn and Peters, 2017). Up to 85% of college students have experienced test anxiety, and approximately 20–25% have experienced high test anxiety (Huntley et al., 2019; Wells et al., 2021). Test anxiety is one of the main reasons college students seek out mental health and support services (Rückert, 2015). Reducing test anxiety has long been an important concern in higher education (Zhang et al., 2022).

Test anxiety is a set of abnormal physiological and psychological phenomena caused by excessive worry about test results before, during, and after the test, manifested as inattention, memory difficulty, confusion, anxiety, and some physical symptoms such as muscle tension, rapid heartbeat, sweating, headache, and gastrointestinal disorder (McCaffrey et al., 2009; Qin et al., 2021).

When test anxiety reaches a certain point, memory and cognition are affected, the brain loses its capacity for clear thought, and learning is hampered (Hjeltnes et al., 2015). These effects might result in academic failure or poor academic performance. Cassady (2004) found that there was a negative correlation between test anxiety and academic performance. Students who suffer from long-term test anxiety will have doubts about their ability and experience self-defeat, self-doubt, and unpleasant emotions, such as fear and helplessness, which may affect their physical and mental health in serious cases (Shapiro, 2014; Badrian et al., 2022). Test anxiety is common in all subjects and intensifies in higher education (Markman et al., 2011). Taking active and effective intervention measures can relieve test anxiety in college students, which is of great significance for the development of individual physical and mental health.

Currently, many studies have used aromatherapy to treat test anxiety in college students, but the outcomes were debatable. Although some studies have shown that aromatherapy is effective in alleviating test anxiety in college students (Ko et al., 2013; Özer et al., 2022), some studies have not (Johnson, 2014; Jafarbegloo and Bakouei, 2020). These results are controversial, and the exact efficacy of aromatherapy has not been fully confirmed. In addition, the relationship between the positive effects of aromatherapy and related factors (such as the type of essential oil, the type of essential oil devices, and the pathway of action of essential oil) are unclear and no standardized clinical practice guidelines have been developed. Additionally, the existing studies have some shortcomings, such as small sample sizes, differences in study design and measures, and producing conflicting results. A meta-analysis addresses these shortcomings by synthesizing the existing studies into one comprehensive study. We hypothesized that aromatherapy could effectively reduce college students’ test anxiety. This meta-analysis aimed to thoroughly review the existing studies and evaluate the effects of aromatherapy on test anxiety in college students to confirm our hypothesis. This study not only provides supporting more scientific and accurate evidence for the application of aromatherapy but also provides new ideas for future research.

## 2. Methods

The study was conducted following the Preferred Reporting Items for Systematic Reviews and Meta-Analyses (PRISMA) guidelines (Moher et al., 2009). The study protocol was registered on PROSPERO (CRD42022345767).

### 2.1. Search strategy

We searched databases including PubMed, The Cochrane Library, Web of Science, Embase, CINAHL, Science Direct, Chinese National Knowledge Infrastructure (CNKI), Chinese Science and Technology Journal Full-Text Database (VIP), and Wanfang Data from inception to June 2022. We combined Medical Subject Headings (MeSH) terms and free-text terms to identify eligible studies. The subject words used were “aromatherapy” and “test anxiety.” Other words were free words. Search terms included the following: aromatherapy, aromatherapies, aroma therapy, aroma therapies, essential oil, aromatic massage, inhalational aroma, test anxiety, exam anxiety, exam stress, and pre-exam anxiety (The specific search strategy of PubMed is shown as an example in Supplementary Table 1).

### 2.2. Inclusion criteria

#### 2.2.1. Participants

The participants were college students.

#### 2.2.2. Intervention and controls

Trials that compared aromatherapy intervention with no intervention or placebo intervention were included, regardless of the form, the type of essential oil, frequency, or duration.

#### 2.2.3. Outcomes

We included studies using test anxiety symptoms as a primary outcome and containing extractable test anxiety scores. Outcomes were measured using validated tools.

#### 2.2.4. Studies

Only randomized controlled trials (RCTs) were eligible.

### 2.3. Exclusion criteria

The following were exclusion criteria: (1) aromatherapy was used in combination with other interventions; (2) animal research; (3) non-full-text articles; (4) incomplete data that could not be obtained by contacting the authors; and (5) overlapping studies.

### 2.4. Study selection

The search results were imported into Endnote software. Studies were screened independently by two researchers using the mentioned inclusion criteria. Any disagreement between the two researchers was settled through conference discussion or, if needed, by a third researcher. After duplicated studies were eliminated, two researchers independently screened the titles and abstracts, excluding studies that did not adhere to the criteria. Then, we went through the entire article one more time to weed out the appropriate studies. In addition, the reference lists of the included studies were used to search for further potentially relevant studies. For information that was not identified but was considered crucial to the study, if needed, we contacted the original study’s authors by phone or email.

### 2.5. Data extraction

Data extraction included basic study information (study title, first author, country, date of publication), study design, study subjects, interventions, control measures, outcome indicators, and outcomes.

### 2.6. Risk of bias assessment

The methodological quality of the included studies was independently assessed by two reviewers using the Cochrane Collaboration tool for assessing the risk of bias (Higgins et al., 2011). The evaluation included random sequence generation, allocation concealment, blinding of participants and personnel, blinding of outcome assessment, incomplete outcome data, selective reporting, and other bias. For each of these domains, studies were rated as having a low, unclear, or high risk of bias.

### 2.7. Data analysis

The meta-analysis was conducted with Review Manager Software (version 5.4). Outcomes were all continuous variables. When different measurement tools were used in each study, standard mean difference (SMD) and 95% confidence intervals (CIs) were used as effect size indicators. When *p* < 0.05, the overall effect was considered statistically significant. *I*^2^ was used to measure the statistical heterogeneity among the studies in each analysis. The fixed-effects model was chosen for analysis when the heterogeneity was low (*p* ≥ 0.05, *I*^2^ ≤ 50%). A random-effects model was used for analysis when the heterogeneity was high (*p* < 0.05, *I*^2^ > 50%), and sensitivity analysis was carried out to assess the stability of the findings by gradually deleting each article of supporting evidence. If heterogeneity was identified, subgroup analyses were conducted on types of essential oil, types of essential oil devices, and types of control groups, considering that these variables might have influences on outcomes. The sensitivity analysis and Egger’s test were performed using Stata 17.0.

## 3. Results

### 3.1. Selection outcomes

**FIGURE 1:**
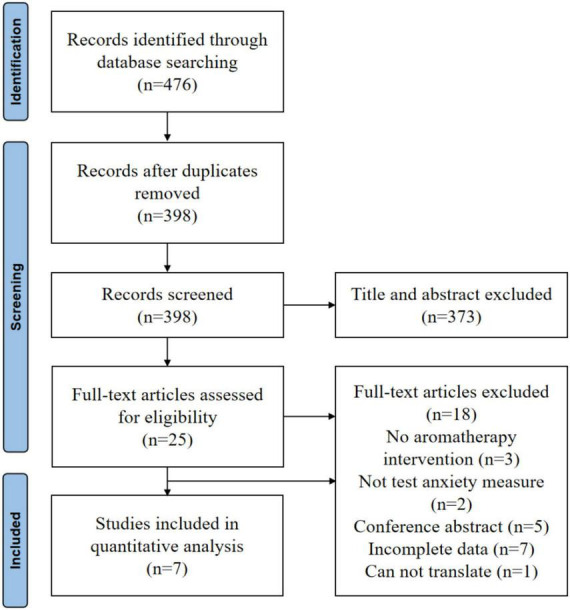
Study selection process.

As shown in Figure 1, a total of 476 studies were screened, 78 of which were duplicated. After two researchers independently assessed the titles and abstracts, 373 studies were excluded. After reviewing the entire text, 18 studies were excluded. Therefore, a total of 7 articles were included in the final meta-analysis (Xie and Jin, 2012; Ko et al., 2013; Johnson, 2014; Bekhradi and Vakilian, 2016; Jafarbegloo and Bakouei, 2020; Hashemi et al., 2021; Özer et al., 2022).

### 3.2. Study characteristics

The characteristics of each included study are displayed in Table 1. The included studies were published between 2012 and 2022. All studies were RCTs, and a total of 425 subjects were enrolled, comprising 217 in the experimental group, and 208 in the control group (blank control group or placebo control group). Three studies (Bekhradi and Vakilian, 2016; Jafarbegloo and Bakouei, 2020; Hashemi et al., 2021) were conducted in Iran, while the other four studies (Xie and Jin, 2012; Ko et al., 2013; Johnson, 2014; Özer et al., 2022) were carried out in Turkey, China, Korea, and the USA. In each study, aromatherapy inhalation was utilized as an intervention. Two studies (Ko et al., 2013; Hashemi et al., 2021) employed a blending of essential oils, three studies (Xie and Jin, 2012; Bekhradi and Vakilian, 2016; Jafarbegloo and Bakouei, 2020) used lavender essential oil, and two studies (Johnson, 2014; Özer et al., 2022) used lemon essential oil. The length of the aromatherapy session varied, while the length of the therapy ranged from a single application to 2 weeks. A variety of measurements were used effectively to assess whether aromatherapy influenced college students’ test anxiety. Three studies (Xie and Jin, 2012; Ko et al., 2013; Jafarbegloo and Bakouei, 2020) used the Test Anxiety Scale (TAI), two studies (Bekhradi and Vakilian, 2016; Özer et al., 2022) used the Sarason Test Anxiety Scale (TAS), one study (Hashemi et al., 2021) used the State-Trait Anxiety Inventory (STAI), and one study (Johnson, 2014) used the Cognitive Test Anxiety Survey (CTAS).

**TABLE 1:** Characteristics of the studies included in the meta-analysis.

Studies	Country	Sample size		Intervention	Control	Essential oil devices	Duration	Measuring tools
		**T**	**C**					
Özer et al., 2022	Turkey	22	24	Lemon essential oil	Blank control	Absorbent	Once	TAS
Hashemi et al., 2021	Iran	35	35	Lavender and damask rose oils	Placebo (sesame oil)	Non-absorbent pad	Once	STAI
Jafarbegloo and Bakouei, 2020	Iran	16	17	Lavender essential oil	Placebo (purified water)	Humidifier	Once	TAI
Bekhradi and Vakilian, 2016	Iran	65	47	Lavender essential oil	Blank control	A box containing several cotton balls	Once a day for 1 week	TAS
Xie and Jin, 2012	China	30	30	Lavender essential oil	Blank control	Pillow	Twice a day for 2 weeks	TAI
Ko et al., 2013	Korea	31	34	A blend of *Acorus calamus*, lavender, and rosewood essential oils	Blank control	Handkerchief	Twice a day for a total of 5 days	TAI
Johnson, 2014	America	18	21	Lemon essential oil	Blank control	Diffuser	Once	CTAS

T, treatment group; C, control group (blank control group or placebo control group); TAI, test anxiety inventory; TAS, test anxiety scale; CTAS, cognitive test anxiety survey; STAI, state-trait anxiety inventory.

### 3.3. Assessment of the risk of bias

**FIGURE 2:**
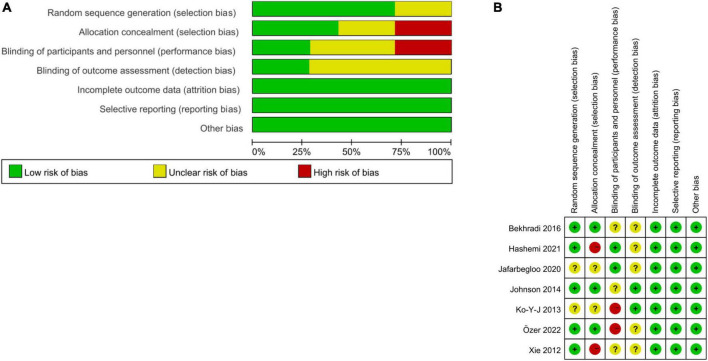
Risk of bias assessment using the Cochrane tool: **(A)** overall trials; **(B)** individual trials.

The risk of bias in the included studies is shown in Figure 2. In multiple studies, some areas were rated as having an “unclear risk of bias” because of the lack of detail reported. All 7 studies were RCTs. Five studies reported the method for random sequence generation (Xie and Jin, 2012; Johnson, 2014; Bekhradi and Vakilian, 2016; Hashemi et al., 2021; Özer et al., 2022), and two studies had not reported (Ko et al., 2013; Jafarbegloo and Bakouei, 2020). Concerning allocation concealment, two studies had a high risk of bias (Xie and Jin, 2012; Hashemi et al., 2021), three studies had a low risk of bias (Johnson, 2014; Bekhradi and Vakilian, 2016; Özer et al., 2022), and the rest had an unclear risk of bias (Ko et al., 2013; Jafarbegloo and Bakouei, 2020). Aromatherapy interventions are difficult for blind participants and outcome assessors, so these two domains had a higher risk of bias. Two studies reported blinding of participants (Jafarbegloo and Bakouei, 2020; Hashemi et al., 2021), two studies did not blind the participants (Ko et al., 2013; Özer et al., 2022), and the rest had an unclear risk of bias (Xie and Jin, 2012; Johnson, 2014; Bekhradi and Vakilian, 2016). Two studies reported blinding of outcome assessment (Ko et al., 2013; Johnson, 2014), while the rest had an unclear risk of bias (Xie and Jin, 2012; Bekhradi and Vakilian, 2016; Jafarbegloo and Bakouei, 2020; Hashemi et al., 2021; Özer et al., 2022). Regarding selective reporting and incomplete outcome data, the risk of bias was low. No other obvious sources of bias were found in the included studies, so the risk of other biases was low. Overall, the risk of bias in the included studies was moderate.

### 3.4. Overall effect

**FIGURE 3:**
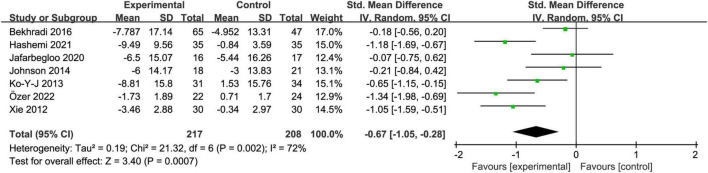
The overall effect of aromatherapy on test anxiety in college students.

Seven trials reported the test anxiety scores of college students before and after the intervention. The *I*^2^ value was 72% (Figure 3), indicating significant study heterogeneity, and thus the random effect model was employed for analysis. According to the findings of this meta-analysis, aromatherapy intervention could effectively relieve the test anxiety of college students [SMD = −0.67, 95% CI: (−1.05, −0.28), *Z* = 3.40, *p* < 0.01] (Figure 3).

### 3.5. Subgroup analysis

#### 3.5.1. Essential oil types

**FIGURE 4:**
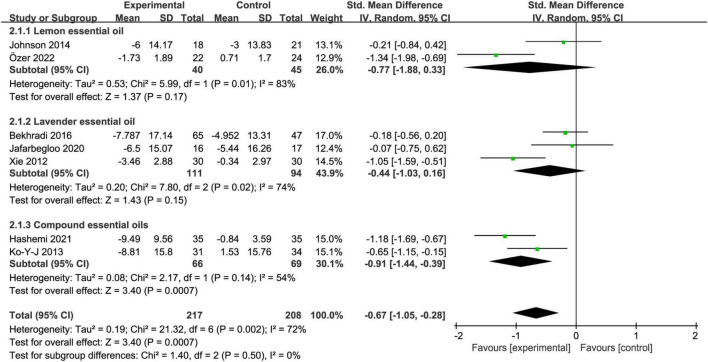
Subgroup analysis of essential oil types.

Aromatherapy intervention with compound essential oils significantly relieved the test anxiety of college students [SMD = −0.91, 95% CI:(−1.44, −0.39), *Z* = 3.40, *I*^2^ = 54%, *p* < 0.01]. Aromatherapy intervention with lemon essential oil [SMD = −0.77, 95% CI:(−1.88, 0.33), *Z* = 1.37, *I*^2^ = 83%, *p* = 0.17] and with lavender essential oil [SMD = −0.44, 95% CI:(−1.03, 0.16), *Z* = 1.43, *I*^2^ = 74%, *p* = 0.15] had no effect on the test anxiety in college students (Figure 4).

#### 3.5.2. Essential oil devices

**FIGURE 5:**
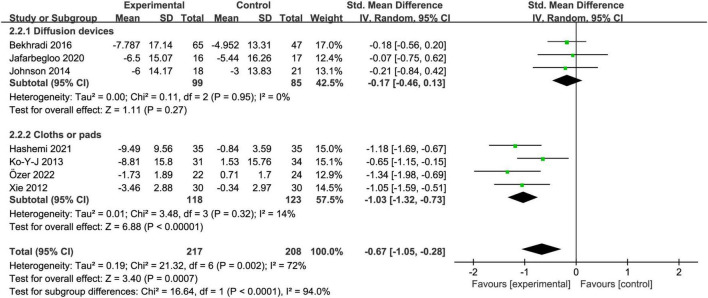
Subgroup analysis of essential oil devices.

Regarding the effects of aromatherapy on test anxiety, two devices used for essential oils were analyzed. Aromatherapy using diffusion devices [SMD = −0.17, 95% CI:(−0.46, 0.13), *Z* = 1.11, *I*^2^ = 0%, *p* = 0.27] had no effect on test anxiety in college students, whereas the use of cloths or pads [SMD = −1.03, 95% CI:(−1.32, −0.73), *Z* = 6.88, *I*^2^ = 14%, *p* < 0.01] as the essential oil devices significantly alleviated test anxiety (Figure 5). In both groups, the *I*^2^ was less than 50%, and heterogeneity decreased significantly.

#### 3.5.3. Control group

**FIGURE 6:**
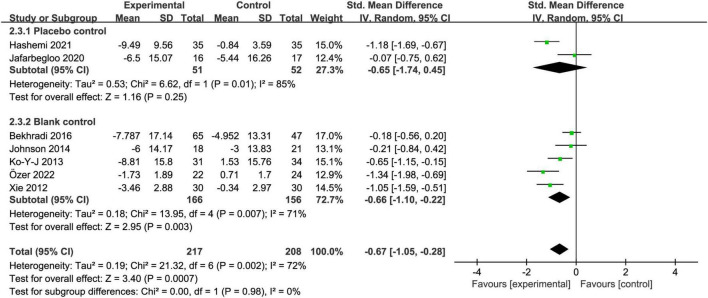
Subgroup analysis of control group types.

Compared with placebo intervention, aromatherapy intervention had no statistically significant effect on test anxiety in college students [SMD = −0.65, 95% CI:(−1.74, 0.45), *Z* = 1.16, *I*^2^ = 85%, *p* = 0.25], but aromatherapy significantly reduced test anxiety compared with blank intervention [SMD = −0.66, 95% CI:(−1.10, −0.22), *Z* = 2.95, *I*^2^ = 71%, *p* < 0.01] (Figure 6).

### 3.6. Publication bias and sensitivity analysis

Because there were fewer than 10 studies included in the meta-analysis, funnel plot analysis was not appropriate. Egger’s test revealed that the included studies had no publication bias (*t* = −0.70, *p* = 0.517). The results of the sensitivity analysis showed that eliminating studies individually did not affect the results, which proves the stability and reliability of the random effect calculation.

## 4. Discussion

Studies on the effects of aromatherapy on test anxiety in college students were comprehensively evaluated in this study, and the quantitative data were analyzed. This meta-analysis, which included seven RCTs, revealed that aromatherapy significantly reduces test anxiety in college students, which is in line with previous studies on the topic (Wells and Hopkins, 2018).

### 4.1. Methodological quality evaluation

The seven studies included in this study were RCTs. The quality of the seven studies was assessed by using the Cochrane risk bias assessment tool, and the results were moderate overall. Seven research studies reported on the comparability of baseline data, and the results were credible. It appeared that the blinding contained the majority of the items with a higher risk of bias. Due to the unique nature of the study, it was challenging to employ the blinding of participants and personnel. It was thought that the lack of the blinding approach had no effect on the resulting bias because the outcome indicators were measured using an objective measurement method. The different essential oil devices used in the included studies were sources of heterogeneity.

### 4.2. Aromatherapy with inhalation is effective

This meta-analysis demonstrated that aromatherapy effectively reduces test anxiety among college students. Test anxiety is a widespread and potentially dangerous problem that makes students question their abilities, which in turn can lead to a lack of concentration and affect academic performance (Sarason, 1975; Wachelka and Katz, 1999). People with test anxiety tend to have higher rates of depression, which suggests its impact on mental health (Herzer et al., 2014). To reduce test anxiety and boost test results among college students, aromatherapy can be utilized as a complementary and alternative therapy. It is easy, safe, and practical. There is a lengthy tradition of using aromatherapy during tests. To improve memory and reactivity, students in ancient Greece carried rosemary wreaths to examination facilities (Zhang, 2012). Aromatherapy can relieve test anxiety through a wide range of physiological and psychological factors. In terms of physiology, essential oils can enter the body *via* the olfactory system, reach the limbic system, and promote the release of neurochemicals such as serotonin and endorphins, which not only have calming and relaxing effects but also stimulate the brain cell response, resulting in enhanced brain storage capability, and memory (Kyle, 2006; Mei, 2010; Faydali and Çetinkaya, 2018; Han et al., 2018). By interacting with the cerebral cortex, the limbic system influences levels of hormones, heart rate, blood pressure, breathing, stress, and blood pressure, producing a calming, relaxing, and anxiety-relieving effect (Karadag and Baglama, 2019). In terms of psychology, there is a significant correlation between odors, memory, and emotions, and when people smell something similar, their memories mind immediately evoked (Wang et al., 2021). Park et al. (2019) discovered in their study that when inhaling the lavender fragrance, subjects felt calm and relaxed.

All the included studies used aromatherapy with inhalation. Aromatherapy with inhalation can have a rapid effect on the human body, triggering a central nervous system response and neurotransmitter release in approximately 4 s (Han et al., 2018). The method of inhalation is not only simple to use but also prevents direct interaction between the essential oil and the body, minimizing the possibility of potential dangers. Aromatic scents are released into the environment using a variety of devices. Aromatic molecules are tiny and volatile. Aromatic molecules enter quickly through the olfactory system, regulate associated neurotransmitters, and impact memory and emotion (Kutlu et al., 2008). Generally, experimental interventions take place either before or during the test. Creating a scented environment can ensure the efficiency of the intervention and help avoid distractions during the exam. Consequently, the method of inhaling the fragrance was more acceptable to the participants.

### 4.3. Compound essential oils are even more effective

The subgroup analysis revealed that aromatherapy with compound essential oils could significantly reduce test anxiety in college students. Lavender and lemon essential oil used alone had no effect on test anxiety, however. This was similar to the finding from a previous study by McCaffrey et al. (2009), which found that lavender and rosemary essential oils combined effectively lowered test anxiety in nursing postgraduates. Lavender essential oil encouraged calmness, while rosemary essential oil helped improve attention and memory. The combined effects of the two essential oils effectively lowered test anxiety. The combination of essential oils increased the number of aromatic molecules and produced synergistic effects, to maximize the curative effect of aromatherapy. Johnson (2019) found that inhalation of lemon essential oil did not relieve cognitive test anxiety in nursing students, which is consistent with our results. In the three studies we included that used lavender essential oil as an intervention, two of them (Bekhradi and Vakilian, 2016; Jafarbegloo and Bakouei, 2020) found that inhalation aromatherapy with lavender essential oil had no effects on students’ test anxiety. The anxiolytic effects of lavender essential oil and lemon essential oil have been reported (Kutlu et al., 2008; Jimbo et al., 2009; Wang and Heinbockel, 2018; Usta et al., 2021; Ou et al., 2022), and we believe that the reason why they do not alleviate test anxiety may be related to the fact that a single essential oil produces fewer anxiolytic effects. Anxiolytic effects can be enhanced by combining them with other essential oils.

### 4.4. Essential oil devices influence the efficacy of aromatherapy

Subgroup analyses showed that aromatherapy using cloths or pads as essential oil devices reduced test anxiety in college students, while aromatherapy using diffusion devices had no effect. After subgroup analysis of essential oil devices, heterogeneity decreased significantly, suggesting that different devices used for essential oils may be potential sources of heterogeneity. It has been reported that the different types of devices used for essential oils may also have an impact on the dosing process as the quality of essential oils may be affected differentially by the devices used (Halligudi and Al Ojaili, 2013). Inhalation of essential oils by dropping them on cloths or pads could ensure the concentration and quality of the essential oils so that aromatic molecules reach brain regions directly and quickly through olfactory pathways (Erdõ et al., 2018).

### 4.5. Does inhaling placebos have the same effect as inhaling aromatic essential oils?

We also conducted a subgroup analysis of the type of control group. We found no statistically significant effect of aromatherapy intervention on test anxiety in college students compared to placebo intervention, but aromatherapy intervention significantly reduced test anxiety compared to the blank intervention. In one (Jafarbegloo and Bakouei, 2020) of the included studies that used pure water as a placebo, it was found that the placebo control group also showed a decrease in TAI scores after the intervention, and there was no significant difference in TAI scores between the placebo and the aromatherapy group after the intervention. This shows that placebos seem to have some effect. Although placebo is not a therapeutic drug, most medical treatment benefits are caused by the brain’s response to the treatment environment (Wager and Atlas, 2015). Therefore, further research is needed to confirm the effect of inhaled aromatic essential oils versus inhaled placebo in alleviating test anxiety.

## 5. Strengths and limitations

This meta-analysis is the first systematic and quantitative overview supporting the clinically and statistically robust effects of aromatherapy on test anxiety in college students. The authors’ evidence may help educators, student mental health workers, students, and their parents resolve test anxiety. This study provides a reference for future research design, in terms of the selection of essential oil types and essential oil devices, as well as the design of research groups. This provides researchers with new ideas and facilitates the generation of new experimental protocols.

This meta-analysis offered a thorough review of the studies on the reduction of test anxiety in college students using aromatherapy, but it still has certain limitations. First, despite a thorough search, there were a relatively small number of studies that matched the inclusion criteria, and the quality of the included studies was only moderate. The gray database was not searched. Second, the outcome measurement tools and interventions in the studies were not completely unified, which may affect the stability of the results. More high-quality and sizable trials, particularly intervention studies, are required in the future to confirm the validity of the results.

## 6. Conclusion

Aromatherapy with inhalation as a new treatment to alleviate test anxiety among college students can be both safe and easy. Compound essential oils can increase the number of aromatic molecules and produce synergistic effects, which are more effective than a single essential oil. Different essential oil devices have different effects, and it is more effective to use cloths or pads as essential oil devices. Further research is needed to confirm the effect of inhaled aromatic essential oils versus inhaled placebo in alleviating test anxiety. Future studies should also consider when they should conduct post-intervention and follow-up evaluations to better assess the impacts of the intervention because test anxiety is context specific. The quantity and quality of the included studies were limited. To confirm the accuracy of the results and provide sensible and effective implementation criteria, more high-quality and sizable trials, particularly intervention studies, are required in the future.

## Author contributions

JL and YZh conceived the presented idea. JL, MY, and ZZ systematically searched and screened the literature. JL, MY, and YZh completed the data extraction. JL and MY performed the meta-analysis. JL and YZh wrote the first draft of the manuscript. JL, YZa, and HC revised the final manuscript. All authors contributed to the article and approved the submitted version.
