# Supplementary material for: Aromatherapy with inhalation effectively alleviates the test anxiety of college students: A meta-analysis
Source: Front Psychol. 2023 Jan 6;13:1042553. doi: 10.3389/fpsyg.2022.1042553 (PMC9853416; doi:10.3389/fpsyg.2022.1042553)
Supplement: Supplementary file 1 [file Data_Sheet_1.PDF]

Supplementary Material

1 Supplementar Tables

Supplementary Table 1. A detailed search strategy for PubMed.

#1	" Aromatherapy "[MeSH Terms] OR " Aromatherapies "[All Fields] OR " Aroma Therapy " [All Fields] OR " Aroma Therapies "[All Fields] OR " Therapies, Aroma "[All Fields] OR " Therapy, Aroma " [All Fields] OR " Essential oil "[All Fields] OR " Aromatic massage "[All Fields] OR " Inhalational aroma " [All Fields]
#2	" Test Anxiety "[MeSH Terms] OR " Anxiety, Test "[All Fields] OR " Exam Anxiety " [All Fields] OR " Anxiety, Exam "[All Fields] OR " Exam Stress "[All Fields] OR " Stress, Exam " [All Fields] OR " Pre-Exam Anxiety "[All Fields] OR " Anxiety, Pre-Exam "[All Fields] OR " Pre Exam Anxiety " [All Fields]
#3	#1 AND #2
